# 768. Digital Patient Education: A Tool to Improve Awareness of CMV

**DOI:** 10.1093/ofid/ofad500.829

**Published:** 2023-11-27

**Authors:** Amy Larkin, Arun S Nair

**Affiliations:** Medscape Education, Nicholasville, Kentucky; Medscape Education, Nicholasville, Kentucky

## Abstract

**Background:**

More than half of US adults over the age of 40 have already been infected by CMV. Despite high prevalence, patient awareness and understanding of CMV is low. This study assessed the impact of online patient education on knowledge, confidence and intent to act regarding CMV.

**Methods:**

The educational intervention consisted of 4 activities hosted on a dedicated learning center on WebMD Education from February-June, 2022. The activities were comprised of text, integrated visuals, and either a patient or healthcare professional (HCP) video commentary. Demographic questions were asked prior to each activity. Knowledge questions were asked both before and after to assess learning gains. Intent to change and confidence questions were asked at the end of each activity. Absolute improvements were calculated for pre/post questions.

**Results:**

Activity 1: **What is Cytomegalovirus (CMV)?**

63,476 learners; 9,239 completers of all questions

72% female; 57% white, non-Hispanic; 18% 35-44 years of age

24% improvement in knowledge regarding complications of CMV infection; 50% intend to talk to HCP about CMV; 52% confident in talking to HCP about what CMV means for family

Activity 2: **CMV: What You Can Do to Help Stop the Spread**

61,818 learners; 3,583 completers of all questions

56% female; 40% black/African American; 22% 25-34 years of age

18% improvement in knowledge regarding the spread of CMV; 75% intend to talk to HCP about CMV; 90% confident in preventing spread of CMV

Activity 3: **Pregnant, Thinking About Pregnancy, or Have a New Baby? What to Know About CMV**

66,918 learners; 4,504 completers of all questions

91% female; 21% black/African American; 44% 25-34 years of age

38% improvement in knowledge regarding CMV in pregnancy and babies; 69% intend to talk to HCP about CMV; 76% confident in understanding how CMV effects pregnancy and babies

Activity 4: **Are You at Risk for CMV?**

2,802 learners; 368 completers of all questions

62% female; 51% white, non-Hispanic; 25% 35-44 years of age

17% improvement in knowledge regarding who is at risk for CMV infection; 61% intend to talk to HCP about CMV; 75% confident in understanding CMV

**Conclusion:**

The outcomes gathered in this assessment are a strong indicator that online patient activities on WebMD Education improved knowledge and confidence and prompted intent to act related to CMV.

**Disclosures:**

**All Authors**: No reported disclosures

